# Supplementary material for: Hybrid Immunity and the Incidence of SARS-CoV-2 Reinfections during the Omicron Era in Frontline Healthcare Workers
Source: Vaccines (Basel). 2024 Jun 19;12(6):682. doi: 10.3390/vaccines12060682 (PMC11209586; doi:10.3390/vaccines12060682)
Supplement: Supplementary file 1 [file vaccines-12-00682-s001.zip › Supplemental Data S1.pdf]

Table S1. Changes in National Methodology for COVID-19 surveillance, regarding Genomic Surveillence

Date of the Methodology	Criteria for selecting the cases in genomic surveillance testing	Criteria for representativity of testing	Point Prevalence Survey in selected ICU wards from the country
02.04.2021	Table 2	No	
03.06.2021	Table 2	No	
02.07.2021	Table 2	No	
23.08.2021	Table 3	No	
22.12.2021	Table 3	No	
06.01.2021	Table 3	No	
11.01.2022	Table 3	No	
20.01.2022	no	Yes	
27.01.2022	no	Yes	
28.01.2022	no	Yes	
01.02.2022	no	Yes	
08.02.2022	no	Yes	
21.02.2022	no	Yes	Yes
18.03.2022	no	Yes	
21.07.2022	Table 4	Yes	
01.08.2022	Table 4	Yes	
22.08.2022	Table 4	Yes	

Table S2. Criteria for selecting cases for testing in the surveillance system, from April 2021 to July 2021

**1.**	Failure to detect the antigenic mark S through RT-PCR in pre-screening
**2.**	Confirmed cases with travel history in countries with new SARS-CoV-2 variants
**3.**	Confirmed cases from areas where there was detected rapid spreading of SARS-CoV-2
**4.**	Direct contacts of cases confirmed with new SARS-CoV-2 variants
**5.**	Direct contacts from clusters with confirmed new SARS-CoV-2 variant
**6.**	Reinfections (minimum 90 days after o previous confirmed case)
**7.**	Vaccine breakthrough infection in vaccinated with the first schedule at minimum detected after 10 days from the last dose

Table S3. Criteria for selecting cases for testing in the surveillance system, from August 2021 to January 2022

**1.**	Failure to detect the antigenic mark S through RT-PCR in pre-screening
**2.**	Confirmed cases with travel history in countries with new SARS-CoV-2 variants
**3.**	Confirmed cases from areas where there was detected rapid spreading of SARS-CoV-2
**4.**	Direct contacts of cases confirmed with new SARS-CoV-2 variants
**5.**	Direct contacts from clusters with confirmed new SARS-C-oV-2 variant
**6.**	Reinfections (minimum 180 days after o previous confirmed case)
**7.**	Vaccine breakthrough infection in vaccinated with the first schedule at minimum detected after 10 days from the last dose

Table S4. Criteria for selecting cases for testing in the surveillance system, from July 2022 to May 2023

**1.**	Direct contacts from clusters with confirmed new SARS-C-oV-2 variant
**2.**	Immunocompromised patients
**3.**	Atypical clinical outcomes
**4.**	Confirmed cases with travel history in countries with new SARS-CoV-2 variants
