# Supplementary material for: Hybrid Immunity and the Incidence of SARS-CoV-2 Reinfections during the Omicron Era in Frontline Healthcare Workers
Source: Vaccines (Basel). 2024 Jun 19;12(6):682. doi: 10.3390/vaccines12060682 (PMC11209586; doi:10.3390/vaccines12060682)
Supplement: Supplementary file 1 [file vaccines-12-00682-s001.zip › Supplemmental Data S2.pdf]

Table S5. Summarised surveillance data regarding sequencing, from reports of the Romanian National Institute of Public Health.

Week	Report's date	Total samples sequenced	Detected VOC	Alpha (B1.1.7 lineage)	Delta variant (B1.1617.2)	Omicron VOC
Week 10.2021	14.03.2021	848	385	381	-	-
Week 11.2021	25.03.2021	1034	546	542	-	-
Week 12.2021	28.03.2021	1050	562	557	-	-
Week 13.2021	04.04.2021	1178	676	670	-	-
Week 14.2021	11.04.2021	1373	863	857	-	-
Week 15.2021	18.04.2021	1502	955	948	-	-
Week 16.2022	25.04.2021	1536	978	970	-	-
Week 17.2023	02.05.2021	1690	1128	1114	-	-
Week 18.2021	09.05.2021	1791	1208	1192	-	-
Week 19.2021	16.05.2021	1854	1267	1248	-	-
Week 20.2021	23.05.2021	1995	1397	1375	-	-
Week 21.2021	30.05.2021	2020	1442	1394	23	-
Week 22.2021	06.06.2021	2087	1490	1437	26	-
Week 23.2021	13.06.2021	2188	1588	1525	35	-
Week 24.2021	20.06.2021	2224	1628	1553	44	-
Week 25.2021	27.06.2021	2307	1711	1621	57	-
Week 26.2021	04.07.2021	2307	1711	1621	57	-
Week 27.2021	11.07.2021	2373	1776	1655	87	-
Week 28.2021	18.07.2021	2415	1817	1682	101	-
Week 29.2021	25.07.2021	2459	1860	1699	127	-
Week 30.2021	01.08.2021	2610	2009	1713	262	-
Week 31.2021	08.08.2021	2663	2061	1714	313	-
Week 32.2021	15.08.2021	2710	2107	1714	359	-
Week 33.2021	22.08.2021	2804	2207	1717	450	-
Week 34.2021	29.08.2021	2976	2373	1719	620	-
Week 35.2021	05.09.2021	3166	2563	1722	807	-
Week 36.2021*	12.09.2021	3516	2913	1722	1157	-
Week 37.2021	19.09.2021	3890	3286	1722	1530	-
Week 38.2021	26.09.2021	4369	3765	1722	2009	-
Week 39.2021	03.10.2021	4860	4255	1722	2499	-
Week 40.2021	10.10.2021	5209	4604	1722	2848	-
Week 41.2021	17.10.2021	5560	4955	1722	3199	-
Week 42.2021	24.10.2021	6022	5417	1722	3661	-
Week 43.2021	31.10.2021	6308	5703	1722	3947	-
Week 44.2021	07.11.2021	6563	5958	1722	4202	-
Week 45.2021	14.11.2021	6896	6291	1722	4535	-
Week 46.2021	21.11.2021	7088	6483	1722	4727	-
Week 47.2021	28.11.2021	7361	6756	1722	5000	-
Week 48.2021	05.12.2021	7524	6918	1722	5160	2
Week 49.2021	12.12.2021	7619	7013	1722	5249	8
Week 50.2021	19.12.2021	7794	7188	1722	5417	15
Week 51.2021	26.12.2021	7891	7285	1722	5504	25
Week 52.2021	02.01.2022	8086	7480	1722	5632	92
Week 01.2022**	09.01.2022	8414	7808	1722	5757	295

Week 02.2022	16.01.2022	8845	8238	1722	5865	617
Week 03.2022	23.01.2022	9156	8549	1722	5895	898
Week 04.2022	30.01.2022	9434	8827	1722	5925	1146
Week 05.2022	06.02.2022	9791	9184	1722	5941	1487
Week 06.2022	13.02.2022	10231	9624	1722	5946	1922
Week 07.2022	20.02.2022	10830	10222	1722	5947	2519
Week 08.2022	27.02.2022	11247	10639	1722	5950	2933
Week 09.2022	06.03.2022	11768	11160	1722	5957	3447
Week 10.2022	13.03.2022	12092	11484	1722	5957	3771
Week 11.2022	20.03.2022	12260	11652	1722	5957	3939
Week 12.2022	27.03.2022	12544	11936	1722	5957	4223
Week 13.2022	03.04.2022	12824	12216	1722	5957	4503
Week 14.2022	10.04.2022	13090	12482	1722	5958	4768
Week 15.2022	17.04.2022	13310	12702	1722	5958	4988
Week 16.2022	24.04.2022	13460	12852	1722	5958	5138
Week 17.2022	01.05.2022	13508	12900	1722	5958	5186
Week 18.2022	08.05.2022	13613	13005	1722	5958	5291
Week 19.2022***	15.05.2022	13770	13162	1722	5958	5448
Week 20.2022	22.05.2022	13936	13327	1722	5958	5613
Week 21.2022	29.05.2022	14075	13466	1722	5958	5752
Week 22.2022	05.06.2022	14158	13547	1722	5958	5833
Week 23.2022	12.06.2022	14251	13639	1722	5958	5925
Week 24.2022	19.06.2022	14388	13776	1722	5958	6062
Week 25.2022	26.06.2022	14447	13835	1722	5958	6121
Week 27.2022	10.07.2022	14766	14154	1722	5958	6440
Week 28.2022	17.07.2022	14937	14325	1722	5958	6611
Week 29.2022	24.07.2022	15222	14610	1722	5958	6896
Week 30.2022	31.07.2022	15500	14888	1722	5958	7174
Week 31.2022	07.08.2022	15753	15141	1722	5958	7427
Week 33.2022	21.08.2022	16277	15660	1722	5958	7946
Week 34.2022	28.08.2022	16624	16006	1722	5958	8292
Week 35.2022	04.09.2022	16871	16253	1722	5958	8539
Week 42.2022	23.10.2022	17762	17138	1722	5958	9424
Week 43.2022	30.10.2022	17780	17152	1722	5958	9438
Week 44.2022	06.11.2022	17892	17260	1722	5958	9546
Week 45.2022	13.11.2022	17941	17309	1722	5958	9595
Week 46.2022	20.11.2022	18041	17383	1722	5958	9669
Week 47.2022	27.11.2022	18041	17383	1722	5958	9669
Week 49.2022	11.12.2022	18176	17470	1722	5958	9756
Week 50.2022	18.12.2022	18190	17474	1722	5958	9760
Week 51.2022	25.12.2022	18255	17518	1722	5958	9804
Week 52.2022	01.01.2023	18274	17525	1722	5958	9811
Week 01.2023	08.01.2023	18360	17566	1722	5958	9852
Week 02.2023	15.01.2023	18411	17591	1722	5958	9877
Week 03.2023	22.01.2023	18513	17618	1722	5958	9904
Week 04.2023	29.01.2023	18597	17642	1722	5958	9928
Week 05.2023	05.02.2023	18597	17642	1722	5958	9928
Week 06.2023	12.02.2023	18746	17688	1722	5958	9974

Week 07.2023	19.02.2023	18746	17688	1722	5958	9974
Week 08.2023	26.02.2023	18792	17690	1722	5958	9976
Week 09.2023	05.03.2023	18835	17708	1722	5958	9994
Week 10.2023	12.03.2023	18946	17708	1722	5958	9994
Week 11.2023	19.03.2023	19038	17708	1722	5958	9994
Week 12.2023	26.03.2023	19210	17708	1722	5958	9994
Week 15.2023	29.03.2023	19487	17708	1722	5958	9994
Week 16.2023	23.04.2023	19555	17708	1722	5958	9994
Week 17.2023	30.04.2023	19632	17708	1722	5958	9994
Week 18.2023	07.05.2023	19632	17708	1722	5958	9994
Week 19.2023	14.05.2023	19683	17708	1722	5958	9994
Week 20.2023	21.05.2023	19835	17708	1722	5958	9994
Week 21.2023	28.05.2023	19858	17708	1722	5958	9994
Week 22.2023	04.06.2023	19893	17708	1722	5958	9994

* Alpha (B1.1.7 lineage) is not representing a VOC
**Omicron becomes dominant in the community
***Omicron BA.2, BA.4 and BA.5 are no longer VOC

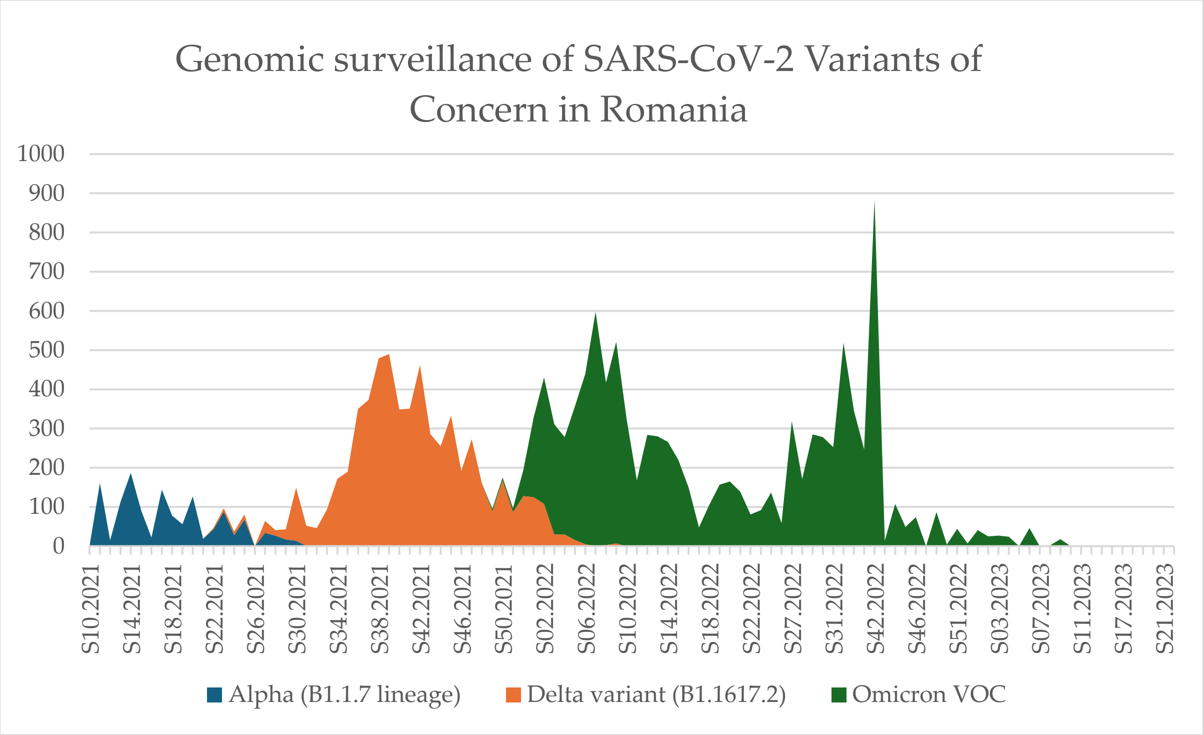

Figure S1. Weekly positive sequenced samples according to reports of the Romanian National Institute of Public Health.
